# Predation on scyphozoan polyps and selective hydrozoan nematocyst incorporation dynamics in an alien aeolid nudibranch

**DOI:** 10.1186/s12983-025-00589-9

**Published:** 2025-11-06

**Authors:** Hila Dror, Tamar Lotan, Dror Angel

**Affiliations:** 1 The Leon Recanati Institute for Maritime Studies and The Department for Maritime Civilizations, University of Haifa, Mt. Carmel, 3103301 Haifa, Israel; 2 Marine Biology Department, The Leon H. Charney School of Marine Sciences, University of Haifa, Mt. Carmel, 3103301 Haifa, Israel

**Keywords:** *Aurelia*, *Cassiopea andromeda*, *Phyllorhiza punctata*, *Rhopilema nomadica*, *Caloria militaris*, *Flabellina affinis*, Jellyfish blooms, Nematocyst, Kleptocnidy

## Abstract

**Background:**

Aeolid nudibranchs prey on various cnidarians and incorporate nematocysts from their prey into the cnidosac, located at the tip of their cerata for self-defense. This study examined the predator–prey interactions between two nudibranch species, *Caloria militaris* and *Flabellina affinis*, and various cnidarians, focusing on scyphozoan polyps from common eastern Mediterranean medusae, including *Aurelia* sp. *Cassiopea andromeda*, *Phyllorhiza punctata*, and *Rhopilema nomadica*. An initial short-term experiment assessed predation by both nudibranch species, after which subsequent experiments focused exclusively on *C. militaris*. Short-term experiments for 24 h and long-term experiments lasting up to 258 days were conducted to determine whether *C. militaris* consumes a variety of cnidarians, and if it incorporates the prey nematocysts into its cnidosacs.

**Results:**

Laboratory experiments indicate that while *F. affinis* avoids scyphozoan polyps, *C. militaris* readily consumes all tested scyphozoan and hydrozoan species and can subsist on them for extended durations of up to 258 days. *C. militaris* predation rate, reaching 95 polyps day^−1^ increased with greater prey availability, but did not reach saturation even at very high prey abundances (> 80 polyps day^−1^), suggesting a higher predation capacity. Surprisingly, despite this intense predation, *C. militaris* did not incorporate any of the scyphozoan nematocysts in its cnidosac; only nematocysts from its known hydrozoan prey were identified in the cnidosac.

**Conclusions:**

*C. militaris* is a generalist that preys on hydrozoa and can extensively feed on a variety of scyphozoan polyps, yet it does not incorporate scyphozoan nematocysts into its cnidosacs. This is the first report to demonstrate complete selectivity in nematocyst sequestration over an extended feeding period in a nudibranch possessing a functional cnidosac. These findings contribute to understanding nudibranch feeding ecology and the potential role these predators may play in regulating jellyfish blooms.

**Supplementary Information:**

The online version contains supplementary material available at 10.1186/s12983-025-00589-9.

## Article highlights

- The nudibranch *Caloria militaris* preys on various scyphozoan polyp species and can subsist exclusively on them for extended periods.
- *C. militaris* feeds on scyphozoan polyps but does not sequester their nematocysts in its cnidosacs.
- Predation on scyphozoan polyps by *C. militaris* may reduce polyp populations and contribute to regulating jellyfish blooms in the eastern Mediterranean.

**Supplementary Information:**

The online version contains supplementary material available at 10.1186/s12983-025-00589-9.

## Background

The eastern Mediterranean has been experiencing massive scyphomedusa blooms during the last several decades, which have negative ecological, economic, and societal effects [1–5]. Scyphozoans typically exhibit a complex life cycle that includes a pelagic medusa stage and a benthic polyp stage. Polyps are considered a primary driver of jellyfish outbreaks since they reproduce asexually and can persist for extended periods, releasing numerous young medusae each season [6, 7]. However, due to their small size and cryptic nature, the location of natural populations of polyps of most Scyphozoa remains unknown. Here we concentrated on Scyphozoa species common to the eastern Mediterranean. These include *Cassiopea andromeda* (Forskål, 1775) (Family: Cassiopeidae), *Phyllorhiza punctata* (von Lendenfeld, 1884) (Family: Mastigiidae), and *Rhopilema nomadica* (Galil, Spanier and Ferguson, 1990) (Family: Rhizostomatidae), which are Indo-Pacific species alien to the eastern Mediterranean [8], whereas *Aurelia* (Lamarck, 1816) (Family: Ulmaridae) is a cosmopolitan genus [9]. Since the 1980’s, *R. nomadica* has become the dominant jellyfish species in the eastern Mediterranean, with populations present throughout most of the year [10]. It is considered one of the "100 worst invasive species" in the Alien Invasive Species Inventories for Europe [11] due to its negative ecological and economic effects and rapid expansion westward [1, 12].

Aeolid nudibranchs (Gastropoda: Nudibranchia) are shell-less marine gastropods that prey on cnidarians such as jellyfish, anemones, and hydroids [7, 13–16]. Many nudibranchs are highly specialized feeders, targeting specific prey, and their larvae settle where prey is abundant [7, 14, 17, 18]. Nudibranchs have evolved defenses against the stinging cells of cnidarians, which contain specialized, syringe-like organelles called nematocysts [19, 20]. These defenses include protective gut linings, specialized epithelial structures, and mucus secretion [13, 21–25]. Through kleptocnidy nudibranchs retain and use their prey’s nematocysts for defense, storing them in cnidosacs at the tips of their cerata (dorsal appendages) [21, 23, 26]. Not all nematocysts are retained, indicating selective sequestration [27–29], which may vary as a result of prey availability [30] and the presence of nudibranch predators [31], with some species preferring to incorporate scyphozoan nematocysts when multiple prey types are available [32].

Typically, selectivity in nematocyst incorporation is manifested as shifts in the relative abundance of nematocyst types between the prey and the cnidosac [25, 33, 34]. For instance, *C. verrucosa* preying on *Aurelia* sp. polyps, incorporated a higher ratio of rhopaloids to a-isorhizas, compared to that present in the polyps [35] and preferentially retained scyphozoan nematocysts over those from other prey species [30].

Kleptocnidy varies widely among nudibranchs, not only in terms of nematocyst selectivity, but also in transfer time, turnover rate, and storage capacity [36, 37]. Transfer may occur within hours (e.g., *Cratena peregrina*) [38] to days (e.g., *Berghia stephanieae* (Valdés, 2005)) [39], and complete turnover within days (e.g., *Spurilla neapolitana*) (Delle Chiaje, 1841) [25] to weeks (e.g., *H. crassicornis*) [40]. These variations highlight species-specific strategies.

The retained nematocysts may persist within the nudibranch's body for extended periods, ranging from days to weeks. Since the inventory of nematocysts (termed cnidome) facilitates taxonomic identification of Cnidaria [41], the presence and composition of nematocysts within the cerata can provide insights into the nudibranch's dietary history, serving as a record of the past and more recently consumed prey [21, 28, 42, 43], as well as the location of these prey.

Jellyfish blooms have significant ecological, economic, and social impacts worldwide [44] with scyphopolyps playing a key role in driving these bloom formations [6, 9, 45]. However, the location of the polyps of most scyphozoan species in situ is unknown. Since this is a major challenge with respect to understanding polyp ecology, many methods (mainly molecular approaches) have been employed to address this problem [42, 46–48]. Mills and Miller [49] were able to identify the prey of the ctenophore *Haeckelia rubra* (Kölliker, 1853) through nematocyst analysis in its predator. Thus, analysis of the nematocysts in the cerata of predator nudibranchs may aid in locating scyphopolyp populations.

**Fig. 1:**
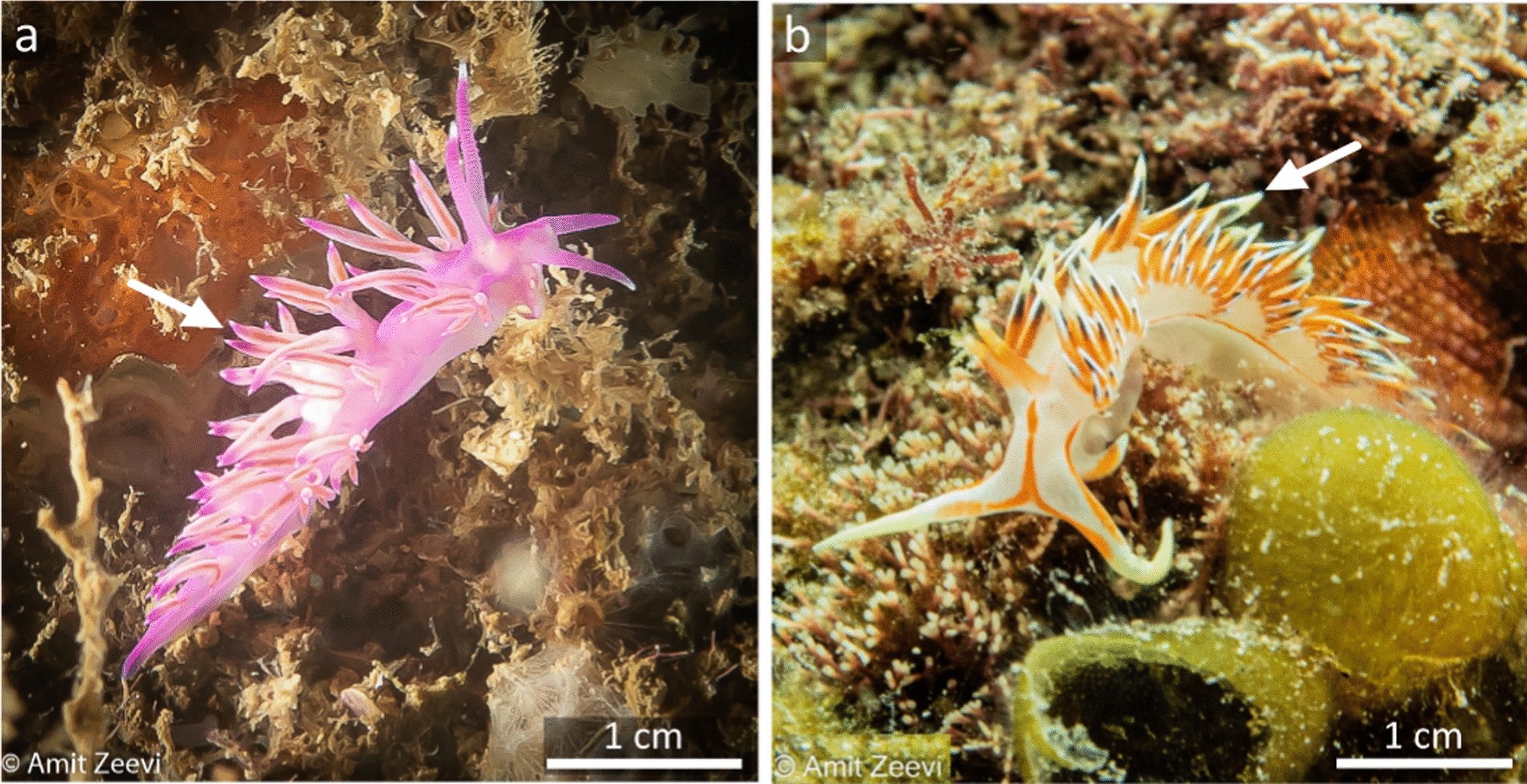
The aeolid nudibranchs **a** *F. affinis* and **b*** C. militaris*. Arrows mark the tips of the cerata. Photos: Amit Zeevi

Here, we focus on two aeolid species, *Flabellina affinis* (Gmelin, 1791) (Family: Flabellinidae) (Fig. 1a)*,* a native aeolid to the Mediterranean, and *Caloria militaris* (Alder and Hancock, 1864) (Family: Facelinidae) (Fig. 1b)*,* a Lessepsian migrant [50]. *F. affinis*, measuring up to 40 mm in length, is present throughout the Mediterranean and along the Atlantic African coasts. It is known to inhabit various habitats including seaweed beds, rocky reefs, and sandy seafloors, up to 10 m deep, and was found to feed on hydroids of the genus *Eudendrium* (Ehrenberg, 1834) [51]*. C. militaris* was originally described in the Bay of Bengal, India, and has been recorded since throughout the Indo-West Pacific [50, 52]. This species is known to inhabit natural hard substrates, although it has also been found on artificial substrates up to 30 m deep [50]. *C. militaris*, measuring up to 40 mm in length, is known to feed on hydroids such as *Calyptospadix cerulea* (Clarke, 1882) (Family: Bougainvilliidae), *Ectopleura larynx* (Ellis and Solander, 1786) (Family: Tubulariidae)*,* and *Pennaria disticha* (Goldfuss, 1820) (Family: Pennariidae) [14, 52]. Along the Israeli Mediterranean coast, *C. militaris* is now commonly found in large numbers during winter and spring.

We examined the interactions between aeolid nudibranchs and cnidarian, focusing on polyps of scyphozoan species commonly found in the eastern Mediterranean. We hypothesized that certain nudibranch species exhibit a more generalist feeding behavior than previously reported, capable of surviving solely on scyphozoan polyps when their known food (Hydrozoa) is scarce. Initial short-term experiments were conducted to compare predation between the two nudibranch species, after which subsequent experiments focused exclusively on *C. militaris* to assess its predation on various cnidarian polyps and to evaluate whether these can sustain the nudibranch as a primary food source over extended periods. In the next step, the incorporation of nematocysts from prey scyphozoan polyps into the cnidosac of *C. militaris* was tested. Several nudibranch species are capable of effectively reducing scyphozoan polyp populations through predation [7, 32, 53–55]. Since polyps represent a key life stage driving jellyfish blooms, their removal may lead to a subsequent decline in bloom formations [56]. Therefore, understanding the feeding behavior of nudibranchs and their predatory impact on scyphozoan polyps is important in our quest to understand the dynamics of scyphozoan populations.

## Methods

### Scyphozoan polyp cultures

Cultures of *Rhopilema nomadica* polyps were produced by laboratory fertilizations of four to eight sexually mature male and female jellyfish collected near Mikhmoret, Israel, eastern Mediterranean Sea (32°24′23″N 34°52′24″E). To enable planula settlement and polyp production, glass microscope slides were placed in a fertilization tank with the adult medusae following our described protocol [57]. *Cassiopea andromeda* medusae and polyps have been maintained in the laboratory since the discovery of their polyps on a rock collected near Jisr az-Zarqa, Israel, eastern Mediterranean (32°53′32″N, 34°90′10″E). Polyps of *Phyllorhiza punctata* were produced after collecting planulae from the oral arms of a gravid female medusa collected near Mikhmoret and allowing them to settle onto glass slides. Polyps of an as yet unidentified *Aurelia* sp. were cultivated to mature medusae and maintained in the laboratory from three polyps that appeared in our flowing seawater aquaria. All scyphozoan medusae were identified based on descriptions from Galil et al. (and references therein) [8]. Each species' polyps (size 0.5–3 mm) were kept in separate containers in a 50 μm filtered flowing seawater aquarium system, maintained at ambient sea surface temperature (SST) ranging annually from 17 to 29 °C. The polyps were fed daily with newly hatched *Artemia salina* (Linnaeus, 1758) nauplii and subjected to a 12-h illumination cycle.

### Nudibranch sampling and maintenance

Between 2020 and 2024, 34 *Caloria militaris* and 4 *Flabellina affinis* nudibranchs were collected by SCUBA diving near Mikhmoret. Identification of the nudibranch species was based on [50] and [58]. The nudibranchs were found on natural rocky (kurkar rock) reefs at depths of 6–12 m during winter and spring (SST 17–23 °C). Each nudibranch was carefully hand-collected into a 50 ml tube and brought to the laboratory for handling. The nudibranchs were kept in 1 L containers of 50 μm filtered flowing seawater, aerated and maintained at SST, subjected to a 12-h illumination cycle. All 34 nudibranchs were used in the feeding experiments, and 13 of these were also used in the nematocyst incorporation experiments.

### Short-term predation of nudibranchs on various cnidarian

Short-term feeding experiments were conducted in 200 ml glass bowls of 50 μm filtered seawater with a glass slide (7.5 × 2.6 cm^2^) holding the prey items. The prey items were chosen from various cnidarian classes based on availability in our laboratory.

The nudibranchs were starved for 72 h, then four *C. militaris* (size 1.5–2.5 cm) and four *F. affinis* (size 1–1.5 cm) individuals were each provided with 13–28 *R. nomadica* polyps (Scyphozoa, size 1–2 mm). In addition, four *C. militaris* individuals (size 1.5–2.5 cm) were each provided with a different cnidarian prey – seven *Exaiptasia diaphana* (Rapp, 1829) (Anthozoa anemone, size 1–2 cm), 14 polyps of *Oculina patagonica* (de Angelis D'Ossat, 1908) (Anthozoa coral, polyp size 1.5–2 mm), eight polyps of *C. andromeda* (Scyphozoa, 2–3 mm), six branches of *Pennaria disticha* (Hydrozoa, polyp size < 1–2 mm) (Supp. Fig. S1). Another set of three *C. militaris* (size 2–3 cm) were first starved for 24 h and then individually introduced into bowls containing three–five 10 cm long tentacles removed from *R. nomadica* medusa (40 cm bell diameter).

The nudibranchs were observed by a Motic SMZ-171 dissecting microscope for three hours to study their feeding behavior, including time to the first encounter with a polyp, response to the encounter, and the manner of feeding. Following this initial close-up examination, the nudibranchs were each placed in an aerated flowing seawater container, maintained at SST, and were allowed to feed for an additional 21 h (overall 24 h) to record predation rate (number of polyps consumed day^−1^), and the predation proportion (number polyps consumed / number polyps provided).

### Long-term predation of *C. militaris* on scyphozoan polyps

The long-term experiments were conducted using only *C. militaris* nudibranchs since *F. affinis* did not prey on scyphozoan polyps in the short-term experiment. Each of the 34 *C. militaris* nudibranchs (sizes 15–30 mm, measured on collection day) were starved for 24 h and then placed in a 1 L container with flowing 50 µm filtered seawater along with polyps of different scyphozoan species on glass slides. In order to decrease size bias, various sizes of nudibranchs were examined for each prey species. The provided prey polyps included *Aurelia* sp., *C. andromeda*, *P. punctata*, and *R. nomadica* (according to availability of polyps at the time of nudibranch collection). To study the predatory capacity of the nudibranchs, polyps were provided at four dose levels per day, from low to very high (≤ 20, ≤ 50, ≤ 80, and > 80 polyps d^−1^, respectively). The polyps were replenished twice weekly, and the predation rate d^−1^, predation proportion d^−1^, and survival period of *C. militaris* were documented. During the experiment all nudibranchs appeared in good condition: feeding continuously, growing, moving, and some laid eggs.

### Review of literature

To better understand the potential effect of predation of *C. militaris* on scyphozoan polyps, we scanned the literature for nudibranch species known to feed on scyphozoan polyps and their reported predation rates. The search was performed during October 2024 using Google Scholar with the search words: nudibranch, predator, scyphozoa, polyp, scyphistoma.

### Prey nematocysts in nudibranch cerata

#### Microscopy and imaging

Examination of cerata and nematocyst samples was performed using Differential Interference Contrast (DIC) microscopy on a Zeiss AXIO Imager.M2 microscope (20X or 40X magnification) and images were captured with a digital Axiocam 503 color camera. Measurements of nematocysts were conducted using a Zeiss Zen-Pro 2.5 program.

#### Identification of incorporated nematocysts in the cnidosac of C. militaris

The nematocyst incorporation experiment was conducted on 13 freshly collected *C. militaris* nudibranchs. Prior to the experiment, 3 to 5 cerata from each of the nudibranchs were removed and stored at −20 °C for identification of nematocysts in nudibranchs collected at sea. Next, the nudibranchs were starved for 96 h and immersed in 5% KCl for 30 s to clear previously-obtained nematocysts from their cnidosacs [59]. After this treatment, cnidosacs in cerata of three nudibranchs were inspected under a microscope to confirm that all nematocysts were ejected.

The nudibranchs were used in two consecutive experiment sets with various prey species (12 days each). Between the first and the second sets, the nudibranchs were treated with 5% KCl to eject previously-incorporated nematocysts from the cerata. In the first set, nudibranchs were provided with polyps of the scyphozoan *Aurelia* sp. (n = 5 nudibranchs) and *C. andromeda* (n = 5 nudibranchs), and the hydrozoan *Nemalecium lighti* (n = 2 nudibranchs). In the second set, nudibranchs were provided with polyps of the scyphozoan *R. nomadica* (n = 6 nudibranchs) and the hydrozoan *P. disticha* (n = 2 nudibranchs) (see supp. Table S1 for details). *P. disticha* was collected from a pier in Jaffa port, Israel (32°03′09″N 34°44′57″E) and *N. lighti* was sampled by SCUBA diving from a 6–12 m deep reef near Mikhmoret. For hydrozoan DNA identification see below. *P. disticha* served as a positive feeding control as *C. militaris* is known to feed on this hydrozoan [14, 52]. Two–three cerata were removed from each nudibranch on the last day of the experiment (day 12) and stored at −20 °C. In addition, for nudibranchs feeding on *Aurelia* sp., *C. andromeda*, and *P. disticha,* cerata were removed from two nudibranchs on day 3, 6, and 9 of the experiment to determine the timeline for nematocyst incorporation into the cnidosac (see supp. Table S1 for sample details). The number of polyps consumed by each nudibranch were counted each time food items were replaced to check that the nudibranchs were actively feeding (supp. Table S2). Squash preparations of the cerata were examined and photographed to check for the incorporation of nematocysts of the various food items in the nudibranch’s cnidosacs. Nematocysts were identified based on the nomenclature proposed by Weill [60] and modified by Östman [41], and the relative abundances of the various nematocyst types in the cnidosacs were documented. For each nematocyst type, width and length of undischarged capsules (n = 10) were measured.

### Nematocyst isolation and characterization from the hydrozoan *P. disticha*

Colonies of the hydrozoan *P. disticha* were sampled from a pier in Jaffa port. Nematocysts were isolated from homogenate of 3–5 branches (2–3 cm long) of *P. disticha* (n = 7) using 50% Percoll (Sigma-Aldrich, USA) followed by centrifugation at 1000 g for 10 min at 4 °C [61]. The resulting pellet was re-suspended in double distilled water (DDW) and stored at −20 °C. Nematocysts discharge was induced by addition of 1 µl of 0.1 M NaOH to 3 µl nematocyst suspensions placed on glass slides. For each sample, the relative abundances of the various types of nematocysts were documented. The identification and description of each nematocyst type based on Östman [41] included measurements of capsule width and length of discharged and undischarged capsules (n = 10). The nematocyst complement of the other prey species used are not presented here because they were not incorporated into the cnidosacs.

### DNA extractions and sequencing of prey Hydrozoa

For the identification of prey hydrozoa collected from the wild, DNA extractions were performed using the DNeasy Blood and Tissue Kit (Qiagen, Hilden, Germany) and the ZymoBIOMICS DNA Miniprep Kit (Zymo Research, USA) following the manufacturer’s protocol. A 600 bp region of the mitochondrial 16S gene was amplified using the primer set SHA/SHB [62]. PCR amplifications were carried out in 25 µl reactions containing 0.5 µl of each primer, (10 µM) 12.5 µl 2X Dream Taq Green Mix (Thermo Scientific), 1 μl BSA (20 mg/ml), 0.5 µl of template DNA (10 ng), and 10 μl of nuclease-free water. Cycling conditions were: 95 °C for 2 min, 35 cycles of 95 °C for 30 s, 50 °C for 30 s, and 72 °C for 1 min with a final extension at 72 °C for 5 min. PCR products were sequenced by "HyLabs" Israel. The taxonomic identities of the sequences obtained were confirmed using BlastN in the NCBI GenBank. Hydrozoan prey species were identified as *P. disticha* and *N. lighti*.

### Data analysis

Data analysis and graphs were performed using RStudio (version 2023.06.0, R Development Core Team 2023). Analysis of variance (ANOVA) was conducted to check for significant differences in predation rate and proportion of polyps consumed between the various scyphozoan polyps offered as food. All tests were conducted at the α = 0.05 significance level. Assumptions for ANOVA were checked using Levene’s test for homogeneity of variance and a Shapiro–Wilk test for normality. Tukey’s HSD tests were conducted for multiple pairwise post-hoc comparisons of means for the significantly different ANOVA results. Pearson’s correlation tests were conducted to assess the relationship between nudibranch predation rate and survival duration; and between number of polyps provided and predation rate. Permutational multivariate ANOVA (PERMANOVA) (9999 permutations) were used to test for significant differences between the cnidome of *P. disticha* and the nematocysts found in the cnidosacs of nudibranchs feeding on *P. disticha*. Permutational multivariate analyses of dispersion (PERMDISP) were conducted to test significant PERMANOVA results, to ensure that results were due to structural differences and not to unequal dispersion among groups. Similarity percentage analyses (SIMPER) were calculated to determine the main contributors to the observed dissimilarity in the cnidomes. Results are presented as % or as mean ± SD.

## Results

### Short-term predation of nudibranchs on various cnidarians

All nudibranchs that were placed in the bowls explored the bottom, sides, water surface, and the slide with polyps. *Flabellina affinis* avoided contact with *Rhopilema nomadica* polyps, and no polyps were consumed during the 24 h experiment (Supp. Fig. S2). In contrast, *Caloria militaris* retracted when its oral tentacles first contacted the *R. nomadica* polyp, as if stung, and either moved away or remained still. To avoid being stung when feeding, the nudibranch elevated its head, and engulfed the polyp from the top, consuming the goblet and leaving the stalk (Supp. Video S1).

During the close-up observation, three out of four *C. militaris* individuals were observed feeding on *R. nomadica* polyps. The first *R. nomadica* polyp was consumed 63 min into the experiment. The nudibranch consumed the goblet within a few seconds, then rested for five minutes until it continued crawling. Twenty-four hours later, between 58% (10/17) and 100% (28/28) of the polyps offered had been consumed by *C. militaris* nudibranchs (19 ± 8.83 polyps d^−1^). However, when *C. militaris* individuals were offered tentacles of adult *R. nomadica* medusae, they kept their distance. Upon contact with tentacles, the nudibranchs would curl up, roll and quickly crawl away, probably due to being stung.

Predation of *C. militaris* on *Cassiopea andromeda* polyps was less efficient than on *R. nomadica* polyps, which are usually smaller. Upon first contact, the polyp attacked the nudibranch causing it to retreat. The first *C. andromeda* polyp was approached only 82 min after *C. militaris* was added to the experimental bowl, and it took nearly 30 min for the nudibranch to fully swallow it. By the end of the 24 h experiment, the nudibranch had consumed only three out of eight *C. andromeda* polyps (37.5%).

In contrast to the behavior of *C. militaris* that was offered scyphozoan polyps, when presented with the hydrozoan, *Pennaria disticha*, the nudibranch began feeding immediately and continued throughout the observation period. The nudibranch would crawl on the hydrozoan branch, reach a polyp, remove it from the stalk, and swallow it whole. The sting of the hydrozoan polyps did not seem to affect the nudibranch as it didn’t retract when coming into contact with the polyp tentacles.

Unlike the hydrozoan and scyphozoan polyps, none of the anemone, *Exaiptasia diaphana* and coral, *Oculina patagonica* polyps were consumed by *C. militaris.* The nudibranch made some attempts to approach the anemone but was severely stung and kept away thereafter.

### Long-term predation of *C. militaris* on scyphozoan polyps

*C. militaris* nudibranchs were offered four species of scyphozoan polyps as prey at four dose levels and a summary of the predation rate, proportion of polyps consumed, and survival duration are provided in Table 1 (see Supp. Table S3 and S4 for details).

**Table 1:** Rate and proportion of tested scyphozoan polyp species (*Aurelia* sp*., C. andromeda, P. punctata,* and *R. nomadica*) consumed by *C. militaris* nudibranchs per day during the long-term feeding experiments

Polyp species	Number of polyps provided d^−1^	N	Predation rate (polyps d^−1^)	Predation proportion	Maximum survival (d)
*Aurelia* sp.	Total	9	8.02 ± 4.69	0.35 ± 0.30	38
	Low (≤ 20)	7	8.21 ± 4.98	0.55 ± 0.30	
	Medium (≤ 50)	6	9.26 ± 5.28	0.29 ± 0.19	
	High (≤ 80)	3	1.83 ± 3.18	0.02 ± 0.03	
	Very high (> 80)	3	13.50 ± 2.29	0.11 ± 0.04	
*Cassiopea andromeda*	Total	17	14.63 ± 12.71	0.68 ± 0.31	258
	Low (≤ 20)	16	6.20 ± 3.97	0.69 ± 0.26	
	Medium (≤ 50)	17	22.86 ± 11.12	0.69 ± 0.32	
	High (≤ 80)	7	33.40 ± 28.00	0.53 ± 0.42	
	Very high (> 80)	7	54.80 ± 37.02	0.54 ± 0.37	
*Phyllorhiza punctata*	Total	4	42.84 ± 10.02	0.87 ± 0.05	39
	Low (≤ 20)	2	15.00 ± 2.36	1.00	
	Medium (≤ 50)	4	35.14 ± 9.56	0.85 ± 0.09	
	High (≤ 80)	3	61.87 ± 1.80	0.88 ± 0.11	
	Very high (> 80)	1	95.20	0.96	
*Rhopilema nomadica*	Total	16	4.67 ± 7.78	0.73 ± 0.18	94
	Low (≤ 20)	16	3.17 ± 2.03	0.73 ± 0.18	
	Medium (≤ 50)	1	31.31	0.89	
	High (≤ 80)	1	67.71	0.98	
	Very high (> 80)	1	83.17	0.92	

Presented as mean ± SD. N = number of nudibranchs in each treatment

Provided polyps: low ≤ 20, medium ≤ 50, high ≤ 80, very high > 80

Each nudibranch was provided with various dose levels during the experiment, thus the total number of nudibranchs (N) at the top row of each prey species is lower than the sum of nudibranchs in the dose treatments

The nudibranchs started feeding almost immediately upon encounter with prey. When examining the predation on the different prey species, the mean (and maximum) number of polyps consumed by the nudibranchs per day was 8.02 ± 4.69, 14.63 ± 12.71, 42.84 ± 10.02, and 4.67 ± 7.78 (23, 130, 105, and 91) for *Aurelia* sp., *C. andromeda*, *Phyllorhiza punctata,* and *R. nomadica*, respectively (Supp. Table S4). The proportion of *Aurelia* sp. polyps consumed was lowest (ANOVA, F_(4,42)_ = 5.8, *p* < 0.005) and the predation rate on *P. punctata* polyps was highest among the prey species tested (ANOVA, F_(4,42)_ = 17.3, *p* < 0.005) (Supp. Fig. S3). Nudibranch survival was longest when feeding on *C. andromeda* polyps (78.8 ± 54.8, max 258 days) and shortest when feeding on *Aurelia* sp. polyps (27.5 ± 7.0, max 38 days) (Supp. Table S4). Generally, survival was longer when the predation rate was higher (Pearson’s correlation, R_(32)_ = 0.52, *p* < 0.005). However, this result was affected mostly by the lower predation rates (< 20 d^−1^) (R_(24)_ = 0.51, *p* < 0.01) and insignificant at higher predation rates (R_(6)_ = − 0.25, *p* > 0.5). Whereas 3,382 *R. nomadica* polyps were consumed during the experiment, the podocysts of *R. nomadica* were left untouched.

When provided with ample food (> 80 polyps d^−1^), the nudibranch consumed between 13 and 95 polyps d^−1^, depending on the prey species. Predation rates of *P. militaris* compared to other nudibranchs species known to feed on scyphozoan polyps are provided in Table 2. The predation rate increased when nudibranchs were provided with increasing numbers of prey (Pearson’s correlation, R_(81)_ = 0.82, *p* < 0.005) but did not reach a plateau. Generally, the nudibranchs did not consume all of the provided polyps even when the provided amount of food was low.

**Table 2:** Nudibranch species that prey on scyphozoan polyps and predation rate (when available)

Nudibranch	Schyphopolyp	Reference	Predation rate (polyps d^−1^) *
**Caloria militaris** (Alder and Hancock, 1864)	*Aurelia* sp., *Cassiopea andromeda, Phyllorhiza punctata, Rhopilema nomadica*	This study	50.20 ± 37.58
	*Aurelia* sp.	This study	13.50 ± 2.29
	*Cassiopea andromeda*	This study	54.80 ± 37.02
	*Phyllorhiza punctata*	This study	95.20
	*Rhopilema nomadica*	This study	83.17
**Ceratodoris plana** (Baba, 1960) (as Okenia plana)	*Nemopilema nomurai, Aurelia coerulea, Rhopilema esculentum*	Tang et al. 2021 [63]	30–118 **
**Coryphella verrucosa** (Sars, 1829)	*Aurelia aurita*	Hernroth and Gröndahl 1985 a, b [7, 56]	200
	*Cyanea capillata*	Gröndahl and Hernroth 1987 [64]	
	*Aurelia*	Östman 1997 [35]	
(as **Flabellina verrucosa**)	*Aurelia*	Frick 2005 [30]	
**Cratena pilata** (Gould, 1870) (as **Coryphella** sp.)	*Chrysaora quinquecirrha*	Cargo and Schultz 1967 [65]	11 polyps in 10 min
	*Chrysaora quinquecirrha*	Vogel 1969 [66]	
	*Chrysaora quinquecirrha*	Oakes and Haven 1971 [54]	
**Cuthona sp.** (Alder and Hancock, 1855)	*Chrysaora quinquecirrha*	Soranno 2016 [67]	
**Dendronotus dalli** (Bergh, 1879)	*Aurelia labiata*	Hoover et al. 2012 [32]	
**Dendronotus rufus** (O’Donoghue, 1921)	*Aurelia labiata*	Kozloff 1983 [68]	
	*Aurelia labiata*	Hoover et al. 2012 [32]	
**Eubranchus spp.**	*Chrysaora quinquecirrha*	Sorrano 2016 [67]	
**Facelina bostoniensis** (Couthouy, 1838) (**as Facelina drummondi**)	*Aurelia aurita*	Thiel 1962 [69]	
**Flabellina fusca** (Bergh, 1894)	*Aurelia labiata*	Hoover et al. 2012 [32]	
**Goniobranchus tinctorius** (Ruppell and Leuckart, 1830) (**as Chromodoris tinctoria**)	*Nemopilema nomurai, Aurelia coerulea, Rhopilema esculentum*	Tang et al. 2021 [63]	
**Hermissenda crassicornis** (Eschscholtz, 1831)	*Aurelia aurita*	Keen 1991 [53]	< 120
	*Aurelia labiata*	Hoover et al. 2012 [32]	8.6–31.3 polyps h^−1^ **
	*Aurelia aurita*	Takao et al. 2014 [55]	43–535 **
**Pleurobranchaea maculata** (Quoy and Gaimard, 1832) (**as Pleurobranchaea novaezealandiae**)	*Aurelia* sp.1	Feng et al. 2017 [70]	393.8
	*Nemopilema nomurai, Aurelia coerulea, Rhopilema esculentum*	Tang et al. 2021 [63]	27–78 **
**Sakuraeolis enosimensis** (Baba, 1930)	*Chrysaora quinquecirrha*	Takao et al. 2014 [55]	8–45 **
	*Aurelia* sp.1	Feng et al. 2017 [70]	26.6
**Sakuraeolis sakuracea** (Y. Hirano, 1999)	*Chrysaora quinquecirrha*	Takao et al. 2014 [55]	26–131 **

^*^Predation rates are provided as polyps day^−1^, unless otherwise stated. **Dependent on body size

### Morphological identification of prey nematocysts in the cnidosac of *C. militaris*

**Fig. 2:**
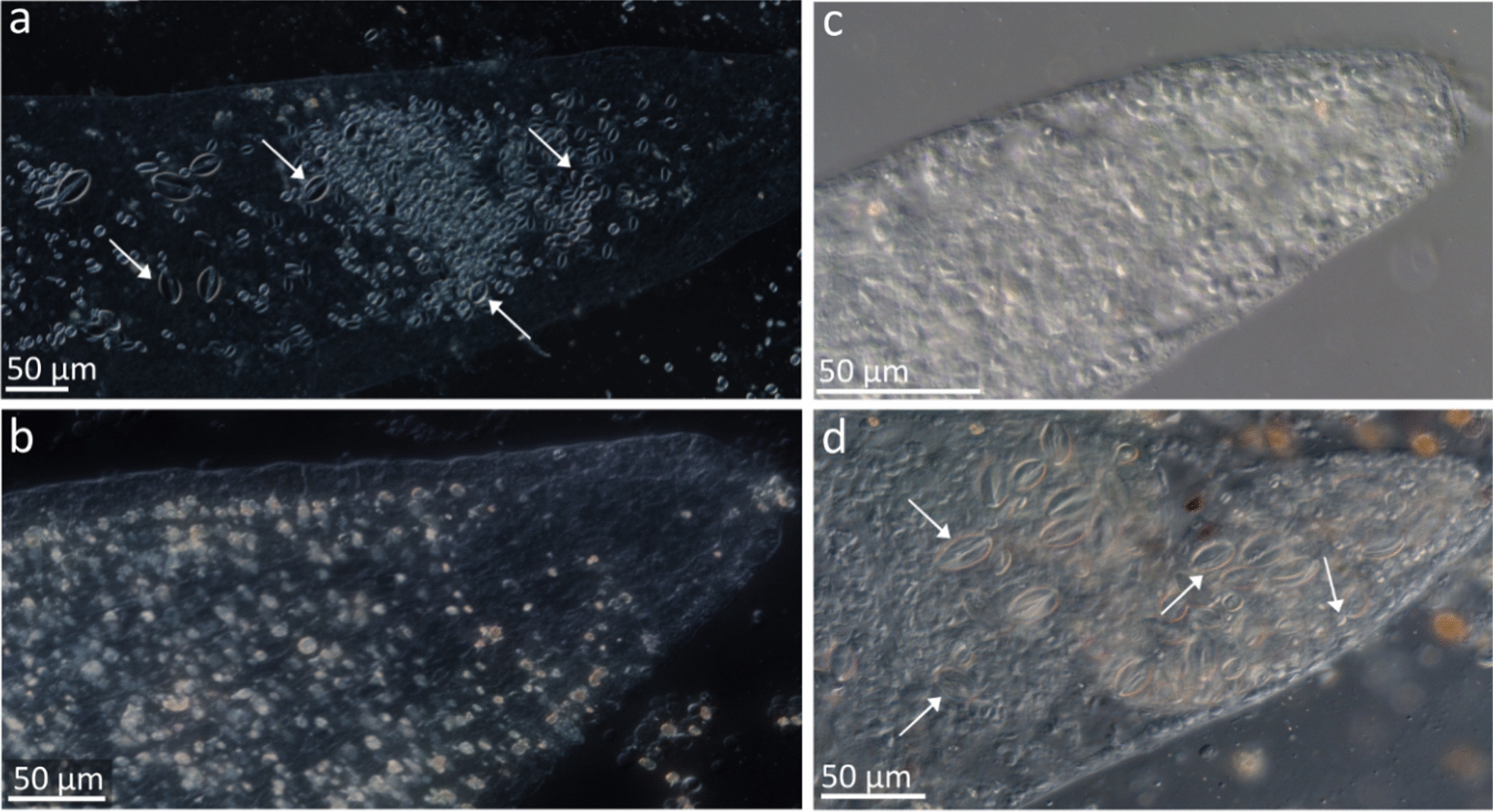
Cnidosacs in the cerata of *C. militaris* nudibranchs. **a** Naturally feeding **b** After treatment with 5% KCl. **c** Fed with polyps of the scyphozoan, *R. nomadica* on day 12. **d** Fed with the hydrozoan *P. disticha* on day 9

*C. militaris* nudibranchs were offered polyps of various Scyphozoa and Hydrozoa as prey, and the incorporation of nematocysts into the cnidosac was examined (see Supp. Table S1 for details)*.* When nudibranchs were immersed in 5% KCl to clear cnidosac from previously incorporated nematocysts, the nudibranchs recoiled and raised their cerata, ejecting a mix of mucus and nematocysts without autotomizing their cerata. Ten minutes after returning to seawater, the animals resumed normal behavior. While the cnidosacs of freshly collected nudibranchs, at sea, contained hundreds of nematocysts (Fig. 2a), most cnidosacs examined after the KCl treatment were completely empty of nematocysts and in others, between 1 and 3 nematocysts were detected (Fig. 2b).

Following cnidosac clearing, *C. militaris* preyed on all prey species provided during the 12-day feeding experiment (supp. Table S2). However, the cnidosacs of nudibranchs that fed on the polyps of *Aurelia* sp., *C. andromeda*, and *R. nomadica*, and on the hydrozoan *Nemalecium lighti* showed no signs of nematocyst incorporation during the experiment at days 3, 6, 9 and 12 (Fig. 2c shows a ceras of *C. militaris* that fed on *R. nomadica* polyps). In contrast, nudibranchs feeding on *P. disticha,* used as a control due to its established role as a natural prey species [14, 52], incorporated the prey nematocysts into their cnidosacs within 3–6 days. Incorporation of hydrozoan nematocysts increased temporally, until by day 9 of the experiment, several hundred hydrozoan nematocysts of various types were concentrated in each of the cnidosacs examined (Fig. 2d).

**Fig. 3:**
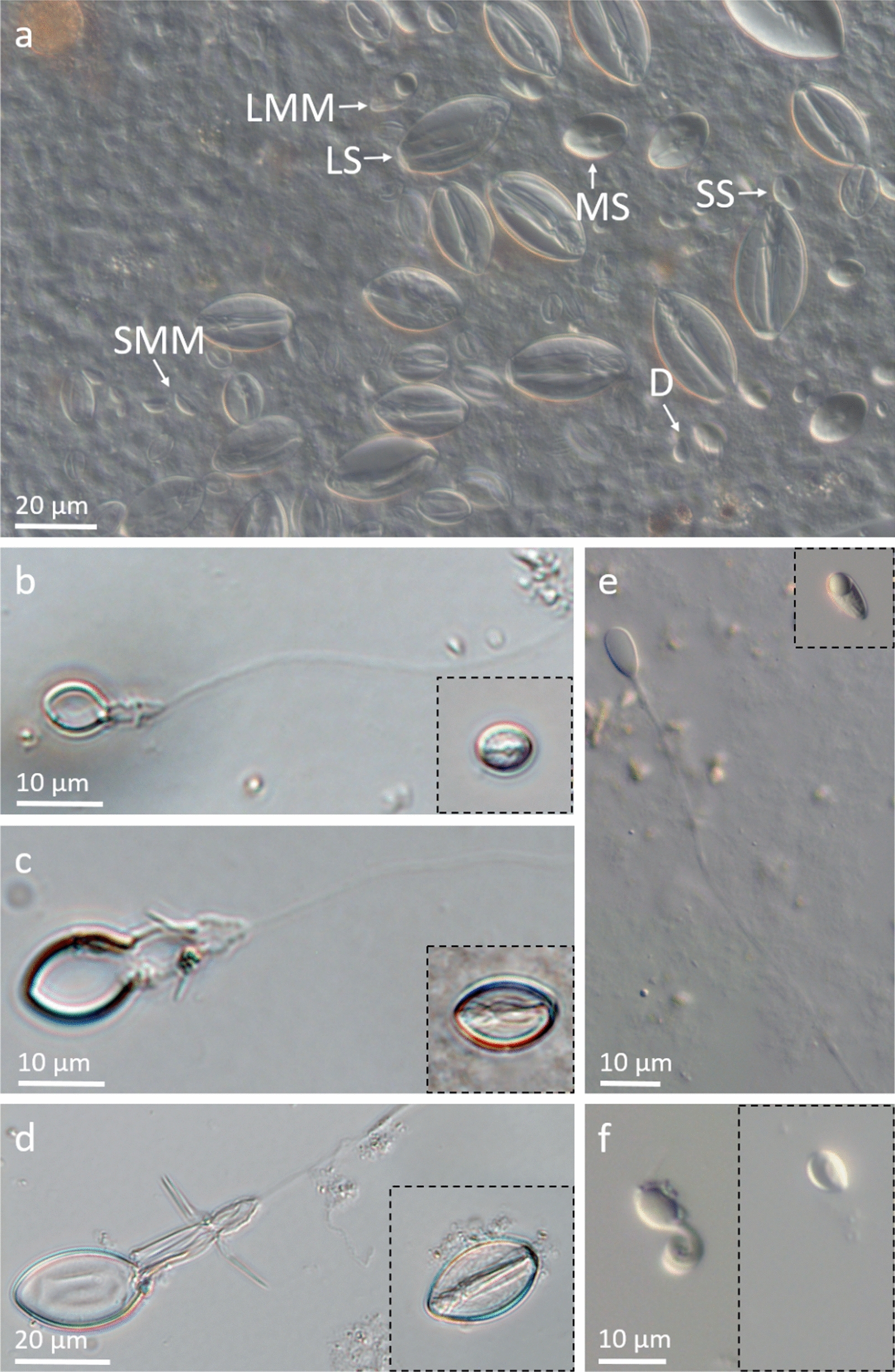
Similar nematocyst types observed in the cnidosacs of *C. militaris* feeding on the Hydrozoa *P. disticha,* and occurring in *P. disticha* **a** Nematocysts in the cnidosacs of *C. militaris* feeding on *P. disticha* (day 9). SS, small stenoteles; MS, medium stenoteles; LS, large stenoteles; LMM, large microbasic mastigophores; SMM, small microbasic mastigophores; D, desmonemes. **b**–**f** Discharged and intact nematocysts types observed in *P. disticha.* **b**–**d** Small, medium, and large stenoteles, **e** Microbasic mastigophores, **f** Desmonemes

The nematocysts found in the cnidosacs of *P. disticha* fed nudibranchs were identified as stenoteles (large, medium, and small), desmonemes, and microbasic mastigophores (large and small) (Fig. 3a). The desmonemes were the most abundant (35.9%), and the large microbasic mastigophores, least abundant (1.5%) (Table 3). The same types and sizes of nematocysts were identified in the hydrozoan, *P. disticha* [71] (Fig. 3b–f, Table 3). However, the relative abundance of the various nematocyst types in *P. disticha* was significantly different than that found in the cnidosacs of nudibranchs fed with this hydrozoan (PERMANOVA, R^2^ = 0.56, *p* < 0.0001). The small stenoteles (Fig. 3a) were the main contributors to the differences observed between the nematocysts in *P. disticha* and the cnidosac samples (SIMPER, 39%); only the desmonemes exhibited similar relative abundances in the cnidosacs and in the Hydrozoa.

**Table 3:** Measurements and relative abundance of the various intact nematocyst types found in the cnidosacs of *C. militaris* nudibranchs after 9 days of preying on the hydrozoan *P. disticha* and in *P. disticha*

		Cnidosacs of *C. militaris*				Hydrozoan *P. disticha*		
Nematocyst	Length (µm)	Width (µm)	Abundance	Relative abundance (%)	Length (µm)	Width (µm)	Abundance	Relative abundance (%)
Small stenotele	7.3 ± 0.1	5.1 ± 0.2	23.1 ± 14.4	18.2 ± 9.8	6.6 ± 0.6	5.2 ± 0.5	73.0 ± 15.9	57.0 ± 10.5
Medium stenotele	13.6 ± 0.8	8.9 ± 0.7	14.6 ± 8.1	11.9 ± 6.6	13.7 ± 1.6	9.5 ± 0.8	5.3 ± 8.5	4.1 ± 6.8
Large stenotele	30.1 ± 2.5	15.9 ± 1.2	22.7 ± 9.5	19.0 ± 8.6	29.0 ± 2.1	16.1 ± 2.3	5.3 ± 8.0	2.3 ± 3.2
Desmoneme	5.3 ± 0.1	3.5 ± 0.1	43.0 ± 16.4	35.9 ± 14.5	5.1 ± 0.2	4.0 ± 0.2	34.6 ± 15.8	27.1 ± 12.5
Small microbasic mastigophores	7.23 ± 0.5	3.8 ± 0.3	15.3 ± 10.3	13.3 ± 10.1	10.1 ± 0.8*	4.5 ± 0.5*	3.0 ± 4.6	2.3 ± 3.7
Large microbasic mastigophores	12.1 ± 0.5	6.3 ± 0.4	1.9 ± 2.3	1.5 ± 2.0	11.1 ± 1.1	6.0 ± 0.5	8.6 ± 4.0	1.8 ± 3.1

Measurements are presented as mean ± SD (n = 10) and relative abundance is presented as percent of > 100 nematocysts counted (n = 7)

^*^Smaller size microbasic mastigophores (n = 6, 7.2 ± 0.9 X 3.0 ± 0.5), similar to the ones found in the cnidosacs were also detected in the hydrozoan samples. However, these were very rare as previously described by Östman [71]

A comparison between nudibranchs in the feeding experiments and wild-caught animals showed that the cnidosacs of wild-caught nudibranchs contained various nematocyst types and sizes (Supp. Fig. S4). Many of the nematocysts found in cnidosacs of wild-caught nudibranchs were small, medium, and large stenoteles (sizes: 5–9 × 4–8 µm, 13–17 × 7–14 µm, and 22–26 × 14–20 µm, respectively), similar in size and shape to the cnidome described above in the cnidosacs of nudibranchs feeding on the hydrozoan *P. disticha.* The most abundant and distinct nematocysts found were bean-shaped microbasic euryteles (ca. 10–16 × 5–8 µm) with a short shaft, divergent from the lateral capsule axis, matching the nematocyst complement of the hydrozoan, *Eudendrium merulum* (Watson, 1985) [72]. Additional nematocysts found only in the cnidosacs of naturally feeding *C. militaris* were round stenoteles (9–12 × 8–11 µm) and small isorhizas (5.5–6.5 × 4–5 µm).

## Discussion

The aeolid nudibranch, *C. militaris,* has previously been reported to feed on several hydrozoan species [14, 52]. Our study showed that *C. militaris* is a generalist and can also prey on various scyphozoan polyps of common eastern Mediterranean species (Supp. videos S1–S3; Table S5), and that these polyps can serve as an exclusive and sustained food source over extended periods.

In the feeding experiments *C. militaris* did not consume all the available polyps even at low prey levels (< 20 polyps d^−1^). The feeding rate of the nudibranch was not constant and appeared to increase with higher food availability. Density-dependent feeding behavior, where predation rate accelerates in response to increased availability of prey, up to a saturation point, has been observed in many invertebrates as well as vertebrates [73, 74]. A similar functional response was observed in the nudibranch *Coryphella verrucosa*, demonstrating density-dependent feeding while not consuming 100% of the prey, even at low prey densities [7, 75]. In our experiments, the predation rate of *C. militaris* did not reach saturation even when provided with > 80 polyps d^−1^. Therefore, the maximum rate of 95 polyps d^−1^ reported here is likely an underestimation of its predatory capacity. The predation rate of *C. militaris* on jellyfish polyps is comparable to rates reported for other nudibranch species (see Table 2), suggesting that it may significantly impact polyp populations in situ through predation [7, 9, 53]. Nevertheless, since these nudibranchs appear to be opportunistic predators [7], and scyphozoan polyp populations may be patchy, predicting the ecological impact of *C. militaris* on polyp populations in the wild remains challenging.

Like other nudibranch species, *C. militaris* does not graze the podocysts of *R. nomadica* [55, 65, 70, 76]. Thus, podocysts may serve as a protective stage, enabling polyp population recovery even after grazers have substantially reduced polyp abundances [77].

While *C. militaris* appeared unaffected by hydrozoan nematocysts, it visibly retracted upon contact with scyphopolyp nematocysts, yet continued feeding. In contrast, contact with anemone nematocysts prompted full retreat. *C. militaris* exhibited a response to scyphozoan polyps attack similar to that of *Cratena pilata*, a known predator of polyps of the scyphozoan *Chrysaora quinquecirrha* (Desor, 1848) in Chesapeake Bay, which has been recorded recoiling upon contact with the polyps’ tentacles yet consuming these nonetheless [65]. These differences in nudibranch responses to prey contact may be influenced by both prey- and predator-related factors. On the one hand, nematocyst toxins vary considerably among prey classes [61]; on the other hand, nudibranchs may secrete prey-specific mucus that inhibits nematocyst discharge [24].

Many aeolid nudibranchs sequester nematocysts from their cnidarian prey for use as defense against predators [13, 21, 23]. This incorporation is selective, influenced by species, prey choice, and predation pressure on the nudibranchs [27, 29, 30, 46, 62–64]. Analysis of cnidosacs from freshly-collected nudibranchs revealed that *C. militaris* exhibits a generalist feeding behavior, such that, in addition to a nematocyst complement consistent with that of the hydrozoans *P. disticha* and *E. merulum* [71, 72], multiple nematocyst types and sizes were observed. Laboratory experiments confirmed that when *C. militaris* fed on *P. disticha*, it retained all of the hydrozoan's nematocyst types in its cnidosac [71]. However, the proportions of these nematocysts differed markedly from those in the hydrozoan itself and the incorporated small-microbasic mastigophores were notably smaller (Table 3). In contrast, the nudibranch *C. pilata* incorporated only microbasic mastigophores from the same hydrozoan [34]. Similar patterns of selective nematocyst incorporation have been documented in many other nudibranch species [25, 27, 29, 30, 33–35, 78]. Our experiments clearly demonstrate that *C. militaris* selectively incorporates nematocysts of specific types or sizes from the hydrozoan *P. disticha*, and when provided with scyphozoan polyps the nudibranch did not sequester any of the prey nematocysts.

Some nudibranchs rely on cnidarian and non-cnidarian prey for different purposes; the non-cnidarian prey provides nourishment and the cnidarian prey a source of nematocysts for defense [32]. In *Phyllodesmium* spp., however, the cnidosac is non-functional and these aeolids rely instead on secondary metabolites from their cnidarian prey rather than incorporating kleptocnides for defense [79]. Here we show, for the first time, that *C. militaris*, despite possessing a functional cnidosac, utilizes scyphozoans exclusively as a food source, disregarding their defensive nematocysts. This distinct strategy of diversified prey consumption yet exclusive nematocyst incorporation is unique among nudibranchs.

*C. militaris* has only recently been introduced into the eastern Mediterranean and is now regularly observed during winter and spring [50]. Therefore, the predation preferences and long-term ecological effects of this species on jellyfish polyp populations require further study. Given the role of scyphopolyps as pivotal contributors to jellyfish proliferations [6], documenting predator–prey interactions between nudibranchs and scyphopolyps is essential to our understanding of medusan population dynamics.

## Conclusions

We found that in addition to feeding on hydrozoans, *C. militaris* preys on a range of scyphozoan polyps including *Aurelia* sp., *C. andromeda*, *P. punctata*, and *R. nomadica,* and can rely solely on these, as food for extended periods of time. Despite its efficient consumption of scyphopolyps, *C. militaris* does not incorporate their nematocysts into the cnidosac, displaying a distinct selectivity in nematocyst sequestration. When prey is abundant, this nudibranch exhibits high predation rates, exceeding 95 polyps d^−1^, indicating its potential to influence jellyfish population dynamics by reducing polyp numbers.

## Supplementary Information

Supplementary Material 1

Supplementary Material 2

Supplementary Material 3

Supplementary Material 4

Supplementary Material 5

## Data Availability

The datasets used and/or analysed during the current study are available from the corresponding author on reasonable request.
