# Supplementary material for: Predation on scyphozoan polyps and selective hydrozoan nematocyst incorporation dynamics in an alien aeolid nudibranch
Source: Front Zool. 2025 Nov 6;22:33. doi: 10.1186/s12983-025-00589-9 (PMC12590869; doi:10.1186/s12983-025-00589-9)
Supplement: Supplementary file 4 — Supplementary Material 4 [file 12983_2025_589_MOESM4_ESM.docx]

**Supplementary material**

**Predation on scyphozoan polyps and selective hydrozoan nematocyst incorporation dynamics in an alien aeolid nudibranch**

Hila Dror^1^, Tamar Lotan^2^, Dror L. Angel^1^*

^1^The Leon Recanati Institute for Maritime Studies, University of Haifa, Mt. Carmel, Haifa 3103301, Israel

^2^ Marine Biology Department, The Leon H. Charney School of Marine Sciences, University of Haifa, Mt. Carmel, Haifa 3103301, Israel

*Corresponding author e-mail: [dangel@univ.haifa.ac.il](mailto:dangel@univ.haifa.ac.il)


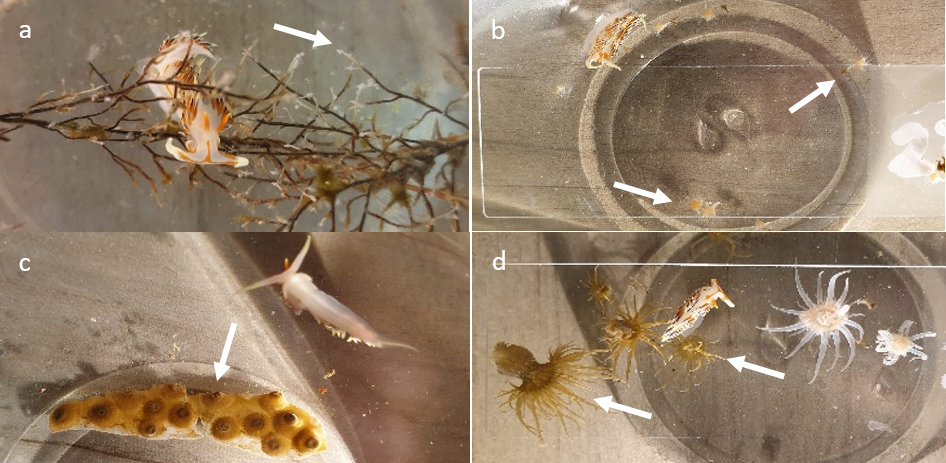


**Supp. Fig. S1**: Experimental glass bowls with various prey species (white arrows) and *C. militaris* nudibranchs. (a) *P. Disticha,* (b) *C. Andromeda,* (c) *O. Patagonica,* (d) *E. diaphana*.


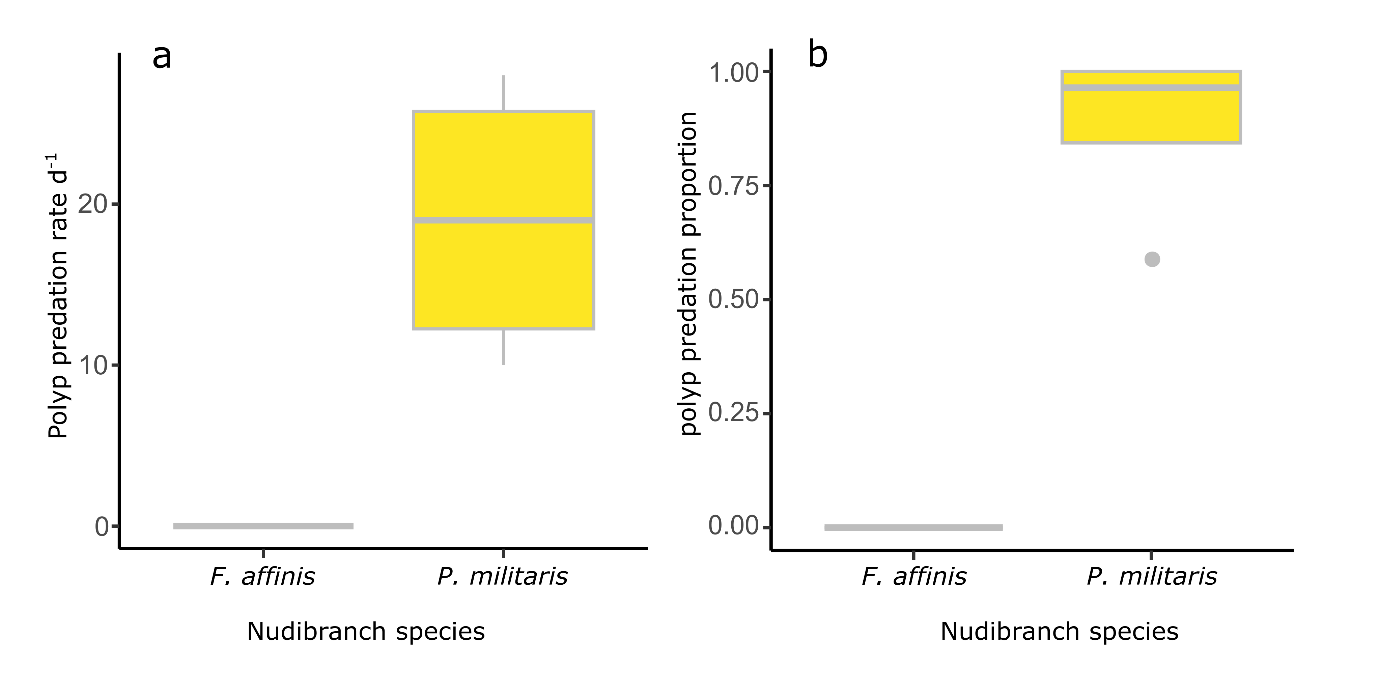


**Supp. Fig. S2**: *R. nomadica* polyps consumed by *F. affinis* (n = 4) and *C. militaris* (n = 4) nudibranchs during the 24 h feeding experiment. (a) predation rate presented as polyps day^-1^ (b) predation proportion (number polyps consumed / number polyps provided).


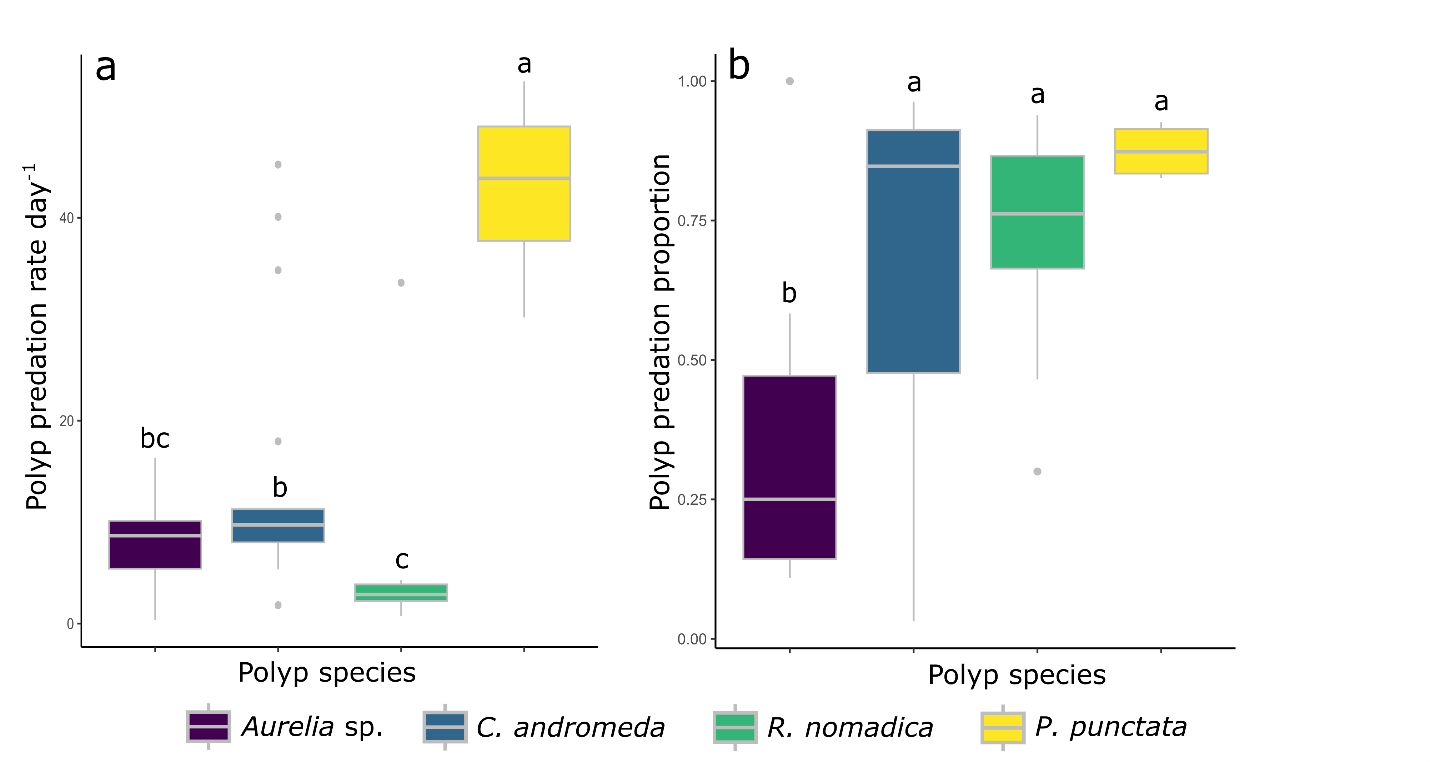


**Supp. Fig. S3**: Predation of *C. militaris* on *Aurelia* sp., *C. andromeda*, *R. nomadica,* and *P. punctata* polyps. (a) Predation rate presented as polyps day^-1^ (b) Predation proportion. Letters indicate significant difference between prey species at P < 0.0001. Number of nudibranchs tested for each prey species: 9, 17, 16, and 4 for *Aurelia* sp., *C. andromeda*, *R. nomadica,* and *P. punctata,* respectively.

**
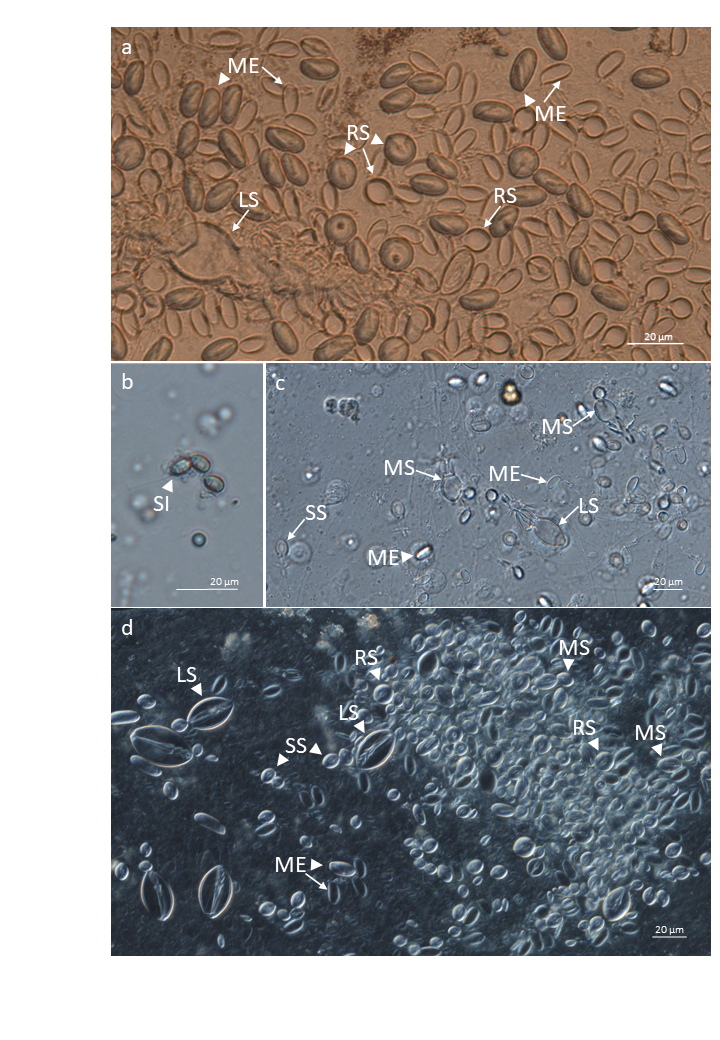
**

**Supp. Fig S4**: Nematocysts from a squash preparation of cerata of a naturally feeding *C. militaris* nudibranch showing discharged (arrows) and undischarged (arrow-heads) nematocysts: Multiple microbasic euryteles (ME, ca. 10-16x5-8 µm), similar to nematocysts of the hydrozoan, *Eudendrium merulum*; small (SS, ca. 5-9x4-8 µm), medium (MS, 17-19x11-14 µm), and large stenoteles (LS, 20-34X14-20 µm) similar to nematocysts of the hydrozoa *Pennaria disticha*; round stenoteles (RS, ca. 9-12x8-11 µm) and small isorhizas (SI, ca. 5.5-6.5x4-5 µm) of an unidentified prey origin.

(a) microbasic euryteles (ME), large (LS) and round (RS) stenoteles, (b) small isorhizas (SI) (c) microbasic euryteles, small (SS), medium (MS), and large stenoteles (d) microbasic euryteles, small, medium, large and round stenoteles.

**Supp. Table S1**: (a) Nudibranch individuals (CM102-114) and prey species used during the 12-day experiment to identify nematocysts in the cnidosac of *C. militaris*. (b): Cerata samples taken from *C. militaris* nudibranchs during the 12-day experiment to identify nematocysts in the cnidosac. Cerata from naturally feeding (Nat) nudibranchs were sampled, then nudibranchs were treated with 5% KCl, and fed Scyphozoa and Hydrozoa polyps (Cas: *C. andromeda*, Au: *Aurelia* sp., Nom: *R. nomadica*, Ap: *A. pluma*, Pd: *P. Disticha*). Each sample consisted of between 2 and 4 cerata (+ indicates cerata samples) excised from each nudibranch.

**Supp. Table S2**: Prey species and predation rate (polyps d^-1^) of *C. militaris* nudibranchs during the 12 d feeding experiment. *C. militaris* individuals are identified as CM with a number. Predation rates presented as mean ± SD (n = 12).

**Supp. Table S3**: Summary of the long-term experiment to examine the predatory capacity of *C. militaris* nudibranchs on four species of scyphozoan polyps (*Aurelia* sp*., C. andromeda, P. punctata*, and *R. nomadica*). Polyps were provided as prey at four doses, from low to very high (≤20, ≤50, ≤80, and >80 polyps d^-1^, respectively) and replenished twice weekly. Individual *C. militaris* nudibranchs are identified as “CM” with a number. *One replicate sample.

**Supp. Table S4**: Summary of the long-term feeding experiment to examine: a) the predatory capacity of *C. militaris* nudibranchs on four species of scyphozoan polyps (*Aurelia* sp*., C. andromeda, P. punctata* and *R. nomadica*) and b) nudibranch survival on these diets. Individual *C. militaris* nudibranchs are identified as “CM” with a number. Number of feeding events is the number of times the indicated prey species was provided. Scyphozoan polyps were replenished twice weekly.

**Supp. Table S5**: Details of the experimental procedures and summary of results.

[**Supp. Video S1**](file:///C:\Users\Hila\OneDrive%20-%20University%20of%20Haifa\PhD\Manuscripts\Nudibranchs\videos\Supp%20video%20S1%20nomadica.mp4): Video capture of *C. militaris* feeding on polyps of *R. nomadica.*

[**Supp. Video S2**](file:///C:\Users\Hila\OneDrive%20-%20University%20of%20Haifa\PhD\Manuscripts\Nudibranchs\videos\Supp%20video%20S2%20cassiopea.mp4): Video capture of *C. militaris* feeding on polyps of *C. andromeda.*

[**Supp. Video S3**](file:///C:\Users\Hila\OneDrive%20-%20University%20of%20Haifa\PhD\Manuscripts\Nudibranchs\videos\Supp%20video%20S3%20aurelia.mp4): Video capture of *C. militaris* feeding on polyps of *Aurelia* sp.
